# Multi-omics reveals metabolic reprogramming underlying differential modulation of nodulation and root development by nitrate and ammonium in soybean

**DOI:** 10.3389/fpls.2026.1813591

**Published:** 2026-04-22

**Authors:** Ruixin Xu, Xiangying Kong, Bingnan Hu, Ming Chen, Chang Wang, Lijuan Qiu, Zhe Yan

**Affiliations:** 1 Key Laboratory of Soybean Molecular Design Breeding, Northeast Institute of Geography and Agroecology, Chinese Academy of Sciences, Changchun, Jilin, China; 2 University of Chinese Academy of Sciences, Beijing, China; 3 The Key Laboratory of Grain Crop Genetic Resources Evaluation and Utilization, Institute of Crop Science, Chinese Academy of Agricultural Sciences, Beijing, China; 4 National Nanfan Research Institute, Chinese Academy of Agricultural Sciences, Sanya, Hainan, China; 5 Xianghu Laboratory, Hangzhou, China; 6 College of Life Science and Technology, Harbin Normal University, Harbin, China; 7 College of Life Sciences and Agri-forestry, Southwest University of Science and Technology, Mianyang, China

**Keywords:** ammonium nitrogen, metabolome, nitrate nitrogen, nodulation and nitrogen fixation, root, transcriptome

## Abstract

**Introduction:**

Nitrogen form and concentration are key environmental regulators that mediate symbiotic nitrogen fixation and root development in legumes.

**Methods:**

To understand the metabolic and molecular mechanisms underlying the effects of distinct nitrogen sources (nitrate and ammonium) on soybean nodulation and root development, this study evaluated root and nodulation phenotypes, and their corresponding transcriptional and metabolomic responses under different concentrations of NH₄Cl or KNO₃.

**Results:**

Results showed that both high concentrations of NH₄Cl and KNO₃ significantly suppressed nodulation and promoted root growth, with nitrate exerting a stronger effect than ammonium. Metabolomic analysis revealed that ammonium treatment enhanced nitrogen assimilation and primary metabolism while suppressing symbiosis-related flavonoids. Nitrate specifically activated chemical defense pathways and inhibited parts of central carbon metabolism. Integrated multi-omics analysis indicated that the nitrogen sources differentially regulated key genes and metabolites involved in nitrogen metabolism, flavonoid/isoflavonoid biosynthesis, and arginine metabolism, leading to distinct metabolic fluxes.

**Discussion:**

Our results demonstrate that soybean perceives different nitrogen forms to orchestrate a metabolic trade-off between autonomous growth, defense, and symbiosis, thereby providing new insights into the mechanistic basis of nitrogen-form adaptation in legumes.

## 1 Introduction

Nitrogen is one of the essential elements for plant growth and development (Leghari et al., 2016). The main forms of nitrogen directly available to plants in soil are nitrate (NO_3_^-^) and ammonium (NH_4_^+^). As the primary inorganic nitrogen source for most terrestrial plants, nitrate is taken up by plants mainly via active transport, followed by a multi-step assimilation process (Albornoz and Godoy, 2025). This pathway involves the sequential reduction of nitrate to ammonium, a highly energy-intensive process that is crucial for converting inorganic nitrogen into organic compounds required for plant growth and development (Liu et al., 2025). Ammonium is readily adsorbed by soil colloids, resulting in low mobility in the soil environment. It can enter plant cells through active transport or passive diffusion, but its capability of long-distance transport within plants is relatively limited (Rather et al., 2025). Unlike nitrate, ammonium can directly participate in assimilation through the glutamine synthetase/glutamate synthase (GS/GOGAT) cycle, thus its assimilation process generally consumes less energy than that of nitrate (Kumari et al., 2022). Under high-concentration stress, excessive nitrate induces nitrite accumulation in plants. Although nitrite is less directly toxic to plants, it exacerbates osmotic stress and promotes reactive oxygen species (ROS) accumulation. In contrast, excessive ammonium tends to cause acidification of root cells and ammonia toxicity, impair the integrity of cell membranes, and inhibit root elongation, thereby negatively affecting plant growth (Zayed et al., 2023).

Soybean (*Glycine max L. Merr.*) is one of the world’s most important crops, serving as a critical source of dietary protein and oil for humans, as well as an indispensable feedstock and industrial raw material (He and Chen, 2013). Different from most plants, legumes can establish a specific symbiotic relationship with rhizobia and form root nodules on their roots, thereby converting atmospheric nitrogen into available ammonium nitrogen through BNF (Geurts et al., 2016). The growth and nodulation of soybean are highly sensitive to nitrogen concentrations. Studies have demonstrated that low concentrations (typically ≤2 mM) of nitrate or ammonium can promote biomass accumulation in soybean (Gulden and Vessey, 1998; Haider et al., 1991; Harper and Gibson, 1984). In contrast, elevated concentrations (e.g., ≥5 mM) may still support plant growth but significantly inhibit nodule formation, development, and nitrogenase activity (Day et al., 1989; Heckmann et al., 1989; Streeter, 1986).

Previous research has partially revealed the genetic and systemic signaling mechanisms underlying the regulation of symbiotic nitrogen fixation by high nitrogen concentration. For instance, nitrate induces the expression of specific CLE peptide genes in Glycine max, Lotus japonicus, and Medicago truncatula, which mediates systemic signaling through shoot-expressed CLE peptide-receptor kinases to suppress nodulation (Lim et al., 2014; Okamoto et al., 2008; Nishida et al., 2016; Lebedeva et al., 2020). At the level of signal perception and transcriptional regulation, nitrate activates NODULE INCEPTION-LIKE PROTEIN (NLP) transcription factors. For example, NLP4 and NLP1 act as master regulators dependent on nitrate, which directly repress symbiotic gene transcription and inhibit nodulation by interacting with the key nodulation factor NIN (Nishida et al., 2021; Misawa et al., 2022; Lin et al., 2018). Different nitrogen forms also exert differential effects on nodulation, with nitrate exhibiting stronger inhibition than ammonium.

Previous omics studies on nitrogen regulation in soybean nodulation and root development have primarily focused on transcriptomic level. However, the metabolic reprogramming induced by different nitrogen sources and its connection to nodulation inhibition and root morphological adaptation remains poorly understood. Metabolomics is a powerful tool for directly profiling metabolic changes in crops under environmental stresses such as salinity, drought, and heat, offering significant advantages in elucidating the metabolic basis of plant adaptation (Li et al., 2019; Fu et al., 2020; Zhu et al., 2022). However, a single-omics approach has inherent limitations. Integrated transcriptomic and metabolomic analysis effectively bridges gene expression and metabolite accumulation, constructing a comprehensive regulatory cascade that spans from signal perception to physiological output, making this approach a powerful tool for dissecting legume pigmentation, seed quality development, and abiotic stress responses (Guo et al., 2024; Xu et al., 2022; Zhang et al., 2023; Zhu et al., 2025; Zhou et al., 2024).

In this study, to elucidate how different nitrogen forms (NH_4_^+^ vs. NO_3_^-^) and concentrations reshape the soybean metabolic network, the transcriptional regulatory architecture underlying these metabolic shifts, and their integrated effects on symbiotic nitrogen fixation and root development, we treated soybean plants with nitrogen−free, high−nitrate, and high−ammonium conditions and systematically performed phenotypic, untargeted metabolomic, and transcriptomic analyses. The multi−omics datasets revealed that nitrate and ammonium impose distinct yet partially overlapping regulatory programs: nitrate caused stronger nodulation inhibition and extensive activation of secondary metabolism and lipid remodeling, whereas ammonium preferentially enhanced nitrogen assimilation and primary metabolic flux. Both nitrogen forms triggered a conserved suppression of flavonoid/isoflavonoid biosynthesis, providing a mechanistic basis for nitrogen−induced inhibition of symbiotic signaling. These integrated findings offer comprehensive insights into the metabolic and molecular mechanisms by which nitrogen availability regulates symbiotic nitrogen fixation and root development in legumes, and provide a conceptual framework for optimizing nitrogen fertilizer management in soybean.

## 2 Materials and methods

### 2.1 Plant growth and treatments

Williams 82 soybean seeds were surface-sterilized (using 100 mL sodium hypochlorite mixed with 4 mL hydrochloric acid), rinsed thoroughly with sterile water, and germinated on sterile, water-moistened filter paper in Petri dishes at 28 °C in the dark for 3 days. Germinated seedlings were transferred to a mixture of vermiculite and perlite (2:1, v/v) and grown in a controlled-climate chamber (28 °C, 16/8 h light/dark cycle, 35% relative humidity).

### 2.2 Rhizobial inoculation and nitrogen treatments

At the time of transplanting, seedlings were inoculated with *Bradyrhizobium diazoefficiens* USDA110. The bacteria were grown on solid YMB medium (0.4 g yeast extract, 10 g mannitol, 0.1 g NaCl, 0.2 g MgSO_4_·7H_2_O, 0.5 g K_2_HPO_4_ per liter) at 28 °C. A single colony was inoculated into liquid YMB medium and cultured with shaking until the OD600 reached 1.0-1.2. Bacterial cells were collected by centrifugation (8000 rpm), washed, and resuspended in N-free nutrient solution to an OD600 of 0.1. Each seedling was inoculated with 10 mL of this suspension applied to the rhizosphere.

Three days after inoculation, nitrogen treatments commenced. Plants were supplied with a modified N-free nutrient solution (containing 0.5 mM MgSO_4_, 0.2 mM CaCl_2_, 0.15 mM K_2_HPO_4_, 1 mM K_2_SO_4_, 0.02 mM FeCl_3_, 0.5 μM H_3_BO_3_, 0.1 μM MnSO_4_, 0.15 μM ZnSO_4_, 0.04 μM CuSO_4_, 2.5 pM NaMoO_4_, 2.5 pM CoCl_2_, and 2.5 pM NiSO_4_) supplemented with either 5 mM NH_4_Cl or 5 mM KNO_3_. Control plants received the N-free solution only. The nutrient solution was replenished every three days.

### 2.3 Phenotyping of root and nodules

To assess the effects of nitrogen treatments on nodulation and root development, plants were harvested three weeks after rhizobial inoculation, coinciding with mature nodule development. Plants were carefully removed from the vermiculite-perlite growth medium, and roots were gently washed to remove adhering substrate. The number of nodules per plant and root fresh weight were recorded. Whole root systems were scanned, and architectural traits—including total root volume, surface area, total length, and average diameter were quantified using the RP Root and Nodule Analysis System (Top Cloud-Agri Technology Co., Ltd.). Statistical significance between each treatment group and the control was evaluated using one-way ANOVA followed by Fisher’s LSD test.

### 2.4 Metabolomic profiling

For metabolomic analysis, root samples were collected 10 days after nitrogen treatments, immediately frozen in liquid nitrogen, and stored at -80 °C. Each treatment group (Control, 5 mM NH_4_Cl or 5 mM KNO_3_) include three biological replicates with each replicate consisting of pooled roots from five individual plants. Non-targeted metabolomic profiling was conducted by Novogene Co., Ltd. using LC-MS.

Raw data were processed using ProteoWizard (v3.0) and XCMS (v3.2) for peak detection, alignment, and quantification. Peak areas were normalized by total ion intensity, and features with >50% missing values were removed. Metabolites with coefficient of variation (CV) < 30% in QC samples were retained.

Metabolite annotation was performed by searching MS/MS spectra against an in-house Novogene database (integrating KEGG, HMDB v4.0, and LIPIDMAPS) with mass error < 5 ppm and matching score > 0.5. Annotation confidence levels followed Metabolomics Standards Initiative (MSI) guidelines.

Differential accumulation analysis was performed using MetaX software. Metabolites with VIP > 1.0 from PLS-DA model, |log_2_FC| ≥ log_2_(1.5) (FC > 1.5 or < 0.667), and P-value < 0.05 (t-test) were considered differentially accumulated metabolites (DAMs). KEGG pathway enrichment analysis was performed using hypergeometric tests (*P* < 0.05).

### 2.5 Transcriptomic profiling

For transcriptomic analysis, root samples were collected concurrently with metabolomic sampling. Total RNA was extracted from the same set of biological replicates (three per treatment, each a pool of five plants). Library preparation and sequencing were performed by Novogene Co., Ltd. on a DNBSEQ-T7 platform.

After quality control, clean reads were aligned to the soybean reference genome (Williams 82) using HISAT2 (v2.0.5). Gene expression levels were calculated as Fragments Per Kilobase of transcript per Million mapped reads (FPKM) using feature counts.

Differential gene expression analysis between treatment groups was conducted using the DESeq2 R package, with genes satisfying |log2(fold change)| > 1 and an adjusted P-value < 0.05 considered differentially expressed genes (DEGs). KEGG pathway enrichment analysis for DEGs was performed using the clusterProfiler R package. Integrated analysis of transcriptomic and metabolomic data was conducted to correlate gene expression changes with alterations in metabolite abundance.

## 3 Results

### 3.1 Ammonium and nitrate mediate differential effects on nodulation inhibition and root morphology

**Figure 1:**
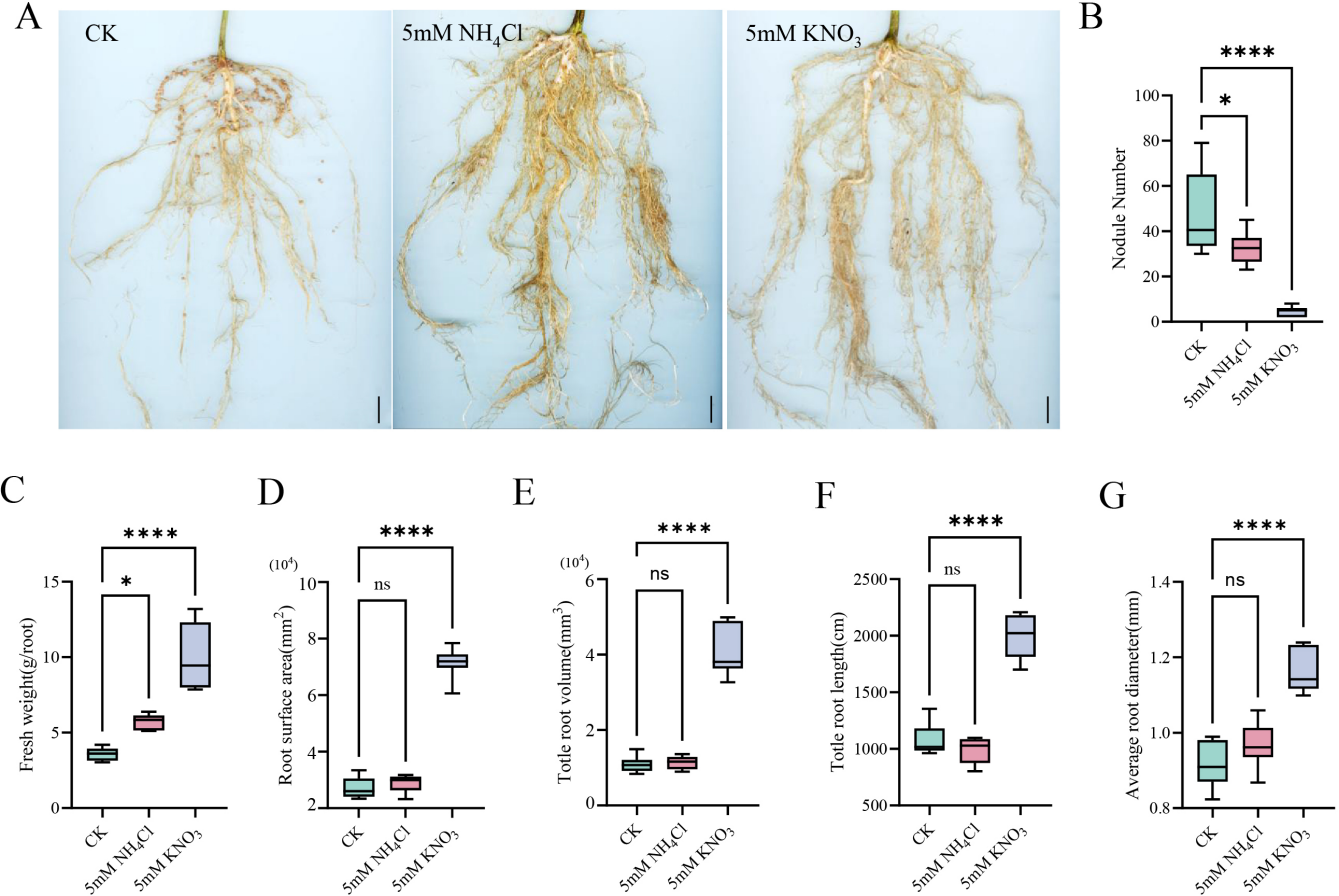
Differential effects of nitrate and ammonium on nodulation inhibition and root development in soybean. **(A)** Root phenotypes of soybean plants under different nitrogen treatments (scale bar = 1 cm). **(B–G)** Nodule number **(B)**, root fresh weight **(C)**, average root diameter **(D)**, total root surface area **(E)**, total root length **(F)**, total root volume **(G)** per plant under control (CK), ammonium (NH_4_Cl), and nitrate (KNO_3_) conditions. Data are presented as mean ± SD (n ≥ 3). Statistical significance was determined by one-way ANOVA followed by Fisher’s LSD test. Asterisks indicate significant differences compared to the control (or between treatments as indicated): *p < 0.05; ****p < 0.0001. “ns” denotes not significant (p ≥ 0.05).

To determine the effects of different nitrogen forms on soybean nodulation, rhizobia-inoculated plants were treated with 5 mM NH_4_Cl, 5 mM KNO_3_, or a nitrogen-free solution (CK). Phenotypic observations revealed that both high-nitrogen treatments significantly suppressed nodulation compared to the CK. Notably, the inhibitory effect of nitrate (KNO_3_) was stronger than that of ammonium (NH_4_Cl), reducing nodule number by 90.4% and 31.6% compared to the CK, respectively. (**Figures 1A, B**).

Beyond inhibiting nodulation, high-nitrogen treatments significantly altered root morphology. While 5 mM NH_4_Cl treatment promoted root biomass, 5 mM KNO_3_ exerted a more pronounced effect, substantially increasing root biomass, average diameter, total surface area, total length, and total volume by 180%, 26.7%, 161.6%, 85%, and 281%, respectively (**Figures 1B–G).** These results demonstrate that high-nitrogen treatments, both nitrate and ammonium, promote root development while inhibiting nodulation, with nitrate exerting more pronounced effects on both symbiosis and root architecture.

### 3.2 Metabolomic profiling of roots under different nitrogen forms

**Figure 2:**
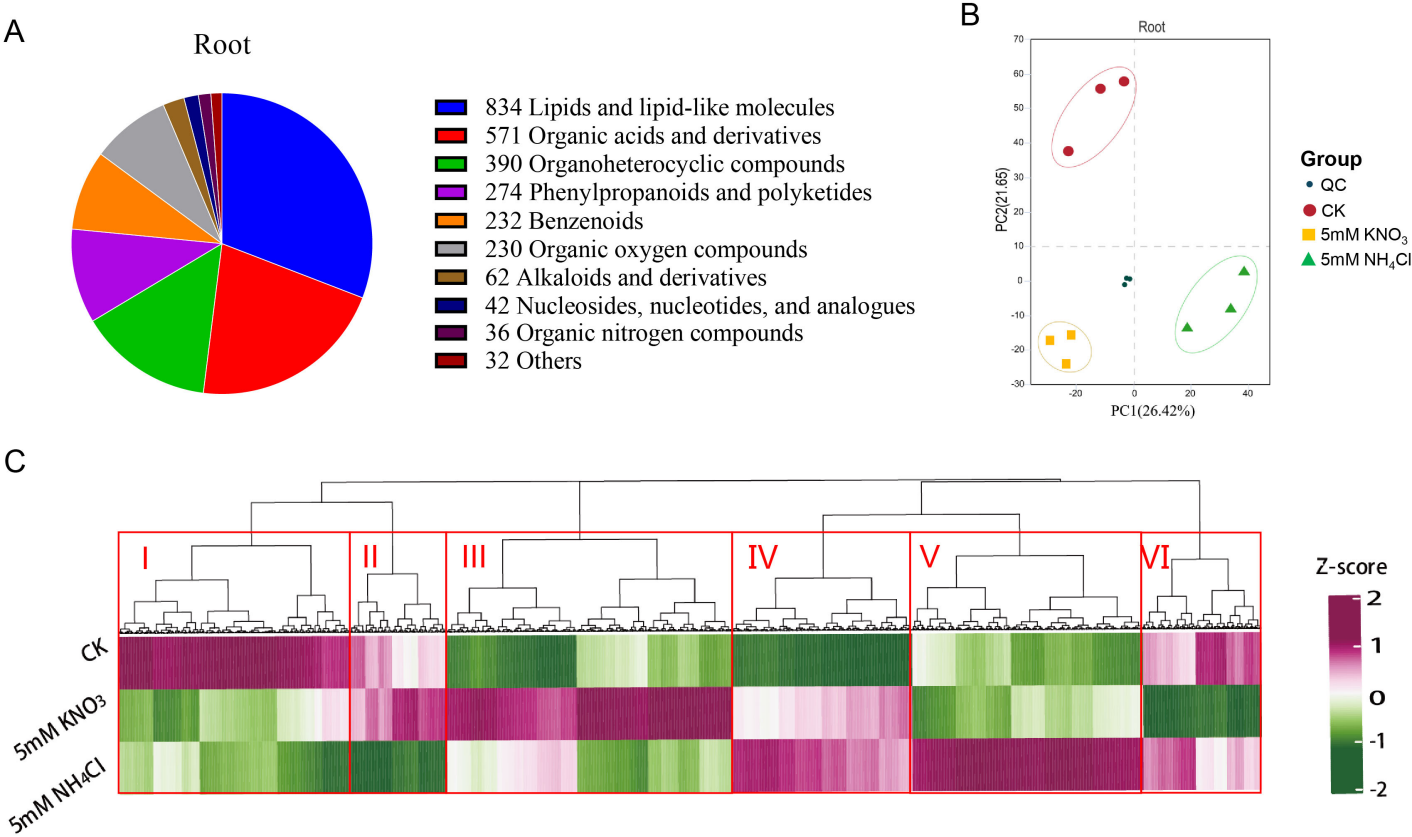
Metabolite profiling in soybean roots under different nitrogen sources. **(A)** Distribution of metabolite categories in roots. **(B)** Principal component analysis (PCA) of metabolic profiles **(C)** Hierarchical clustering analysis of differentially accumulated metabolites in soybean roots under different nitrogen form.

To elucidate the impact of nitrogen form on metabolite accumulation in soybean roots, we performed non-targeted metabolomic analysis on root samples from three groups: CK, 5 mM KNO_3_, and 5 mM NH_4_Cl, each with three biological replicates. A total of 2703 metabolites were identified, primarily belonging to lipids and lipid-like molecules (834), organic acids and derivatives (571), organoheterocyclic compounds (390), phenylpropanoids and polyketides (274), benzenoids (232), and organic oxygen compounds (230) (**Figure 2A**).

Principal Component Analysis (PCA) of chromatographic data from all experimental and quality control (QC) samples showed low variation among QC replicates, indicating stable data quality. The first two principal components, PC1 and PC2, explained 26.42% and 16.65% of the total variance, respectively. Biological replicates within each treatment clustered tightly, while clear separation was observed between different treatment groups, indicating that nitrogen form significantly altered the global metabolite composition of roots (**Figure 2B**).

To systematically analyze the global regulatory features of ammonium and nitrate on the soybean root metabolic network, we performed hierarchical clustering analysis on the non-targeted metabolomic data from the three groups (CK, 5 mM NH_4_Cl, 5 mM KNO_3_). The heatmap revealed significant divergence in root metabolic profiles induced by different nitrogen sources, presenting a clear pattern of treatment-specific and modular regulation of metabolism (**Figure 2C**). Based on the row clustering dendrogram, differentially accumulated metabolites (DAMs) were categorized into six core functional modules: (I) metabolites inhibited by both nitrogen forms; (II) metabolites specifically inhibited by ammonium; (III) metabolites specifically induced by nitrate; (IV) metabolites promoted by both nitrogen forms; (V) metabolites specifically induced by ammonium salts; and (VI) metabolites specifically inhibited by nitrate (**Figure 2C**). These modules were further classified into nitrogen-form-insensitive (Modules I and IV, where metabolites were consistently regulated by both ammonium and nitrate) and nitrogen-source-sensitive categories (Modules II, III, V, and VI, where metabolites were differentially regulated by ammonium and nitrate) (**Figure 2C**).

### 3.3 Functional analysis of DAMs in response to different nitrogen sources

To investigate metabolite accumulation patterns under different nitrogen treatments, we identified and compared DAMs in roots. Compared to CK, 5 mM NH_4_Cl treatment resulted in 611 DAMs (422 up, 189 down). The 5 mM KNO_3_ treatment yielded 635 DAMs (401 up, 234 down) compared to CK. A comparison between 5 mM NH_4_Cl and 5 mM KNO_3_ treatments identified 462 DAMs (270 up, 192 down) (**Supplementary Figure 1**). Venn diagram analysis showed that there were 300 DAMs (234 up-regulated and 66 down-regulated) in response to both nitrogen sources, 140 DAMs (82 up-regulated and 58 down-regulated) specifically responsive to NH_4_Cl, and 128 DAMs (42 up-regulated and 86 down-regulated) specifically responsive to KNO_3_ (**Supplementary Figure 2**).

**Figure 3:**
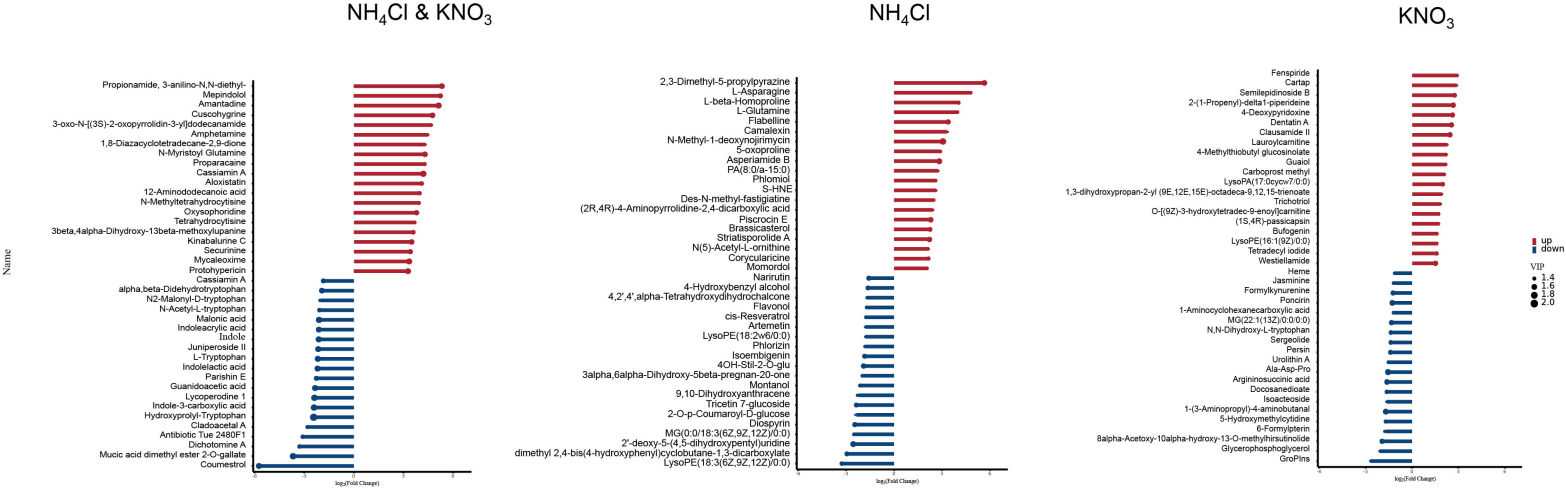
Stem of the top 20 differentially accumulated metabolites induced by ammonium and nitrate. DAMs were defined by VIP > 1.0 and |log_2_FC| ≥ log_2_(1.5). Red bars: significantly upregulated (log_2_FC ≥ 0.585); blue bars: significantly downregulated (log_2_FC ≤ −0.585). Dot size represents VIP value (1.4–2.0). X-axis: log_2_(Fold Change); Y-axis: metabolite names.

A comparison of the top 20 most altered DAMs reveals distinct metabolic responses to ammonium versus nitrate sources (**Figure 3**). Analysis of DAMs responding to both nitrogen sources revealed that commonly upregulated metabolites were primarily enriched in alkaloid biosynthesis (e.g., Cuscohygrine, Securinine) and nitrogenous organic compound modification pathways. Commonly downregulated metabolites were significantly enriched in tryptophan metabolism and its derivatives (e.g., L-Tryptophan, various indole compounds) and coumestans (e.g., Coumestrol) (**Figure 3**). This “up-down” metabolic pattern suggests that high nitrogen supply generally shifts metabolic resources away from the tryptophan-coumestan pathway (associated with auxin synthesis and symbiotic signaling) towards more universal alkaloid defense and nitrogen storage pathways.

DAMs specifically responding to ammonium displayed a distinct pattern related to nitrogen assimilation, defense, and symbiosis. Specifically, ammonium strongly induced the accumulation of key nitrogen assimilation metabolites (e.g., L-Glutamine, L-Asparagine) and direct defense-related compounds (e.g., the phytoalexin camalexin, the oxidative stress marker S-HNE). Concurrently, the synthesis of a series of flavonoids/isoflavonoids involved in rhizobial recognition and nodulation signaling (e.g., Narirutin, Phlorizin) was significantly suppressed (**Figure 3**).

In contrast, nitrate induced a unique metabolic reprogramming related to secondary metabolism, lipid remodeling, coupled with suppression of some primary biosynthesis. Nitrate broadly upregulated defensive secondary metabolites, including various alkaloids (e.g., Fenspiride, Cartap), terpenoids (e.g., Guaiol), glucosinolates (e.g., 4-Methylthiobutyl glucosinolate), and cyclic peptides (e.g., Westiellamide), while promoting lipid metabolic remodeling. Conversely, some core metabolic intermediates (e.g., Heme, Argininosuccinic acid) were downregulated (**Figure 3**). These results suggest that nitrate, potentially acting as a redox and energy signal, preferentially drives the construction of a diversified chemical defense system and lipid metabolism remodeling, while adjusting primary metabolic flux, forming its specific physiological regulatory basis.

Together, these metabolic alterations provide mechanistic insights into the contrasting phenotypes: suppression of flavonoid/isoflavonoid pathways likely weakens rhizobial signaling and nodule initiation, whereas enhanced nitrogen assimilation and defense−related metabolites may redirect resources toward root growth rather than symbiosis.

### 3.4 KEGG pathway enrichment analysis of DAMs from different nitrogen sources

**Figure 4:**
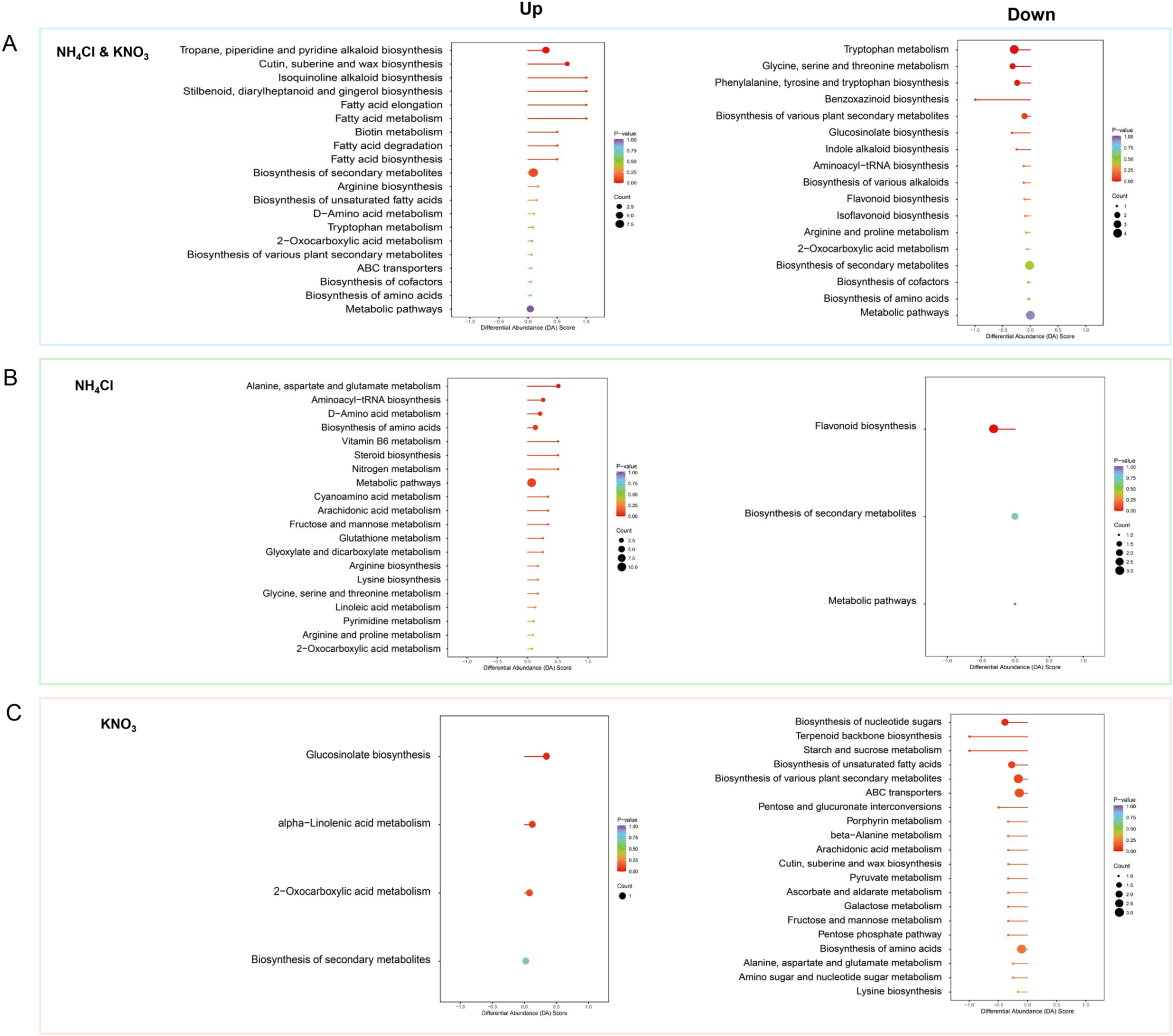
KEGG pathway enrichment analysis of DAMs in soybean roots under different nitrogen treatments. Pathway enrichment of DAMs across three comparisons: **(A)** 5 mM NH_4_Cl vs. 5 mM KNO_3_, **(B)** 5 mM NH_4_Cl vs. CK, **(C)** 5 mM KNO_3_ vs. CK. Up/down panels show pathways enriched by upregulated/downregulated metabolites. X-axis: enrichment factor; Y-axis: pathway names. Dot color = P-value, dot size = number of DAMs.

KEGG pathway enrichment analysis was performed on DAMs responding to both nitrogen sources, as well as those specifically responding to ammonium or nitrate (**Supplementary Figure 3**, **Figure 4**).

Analysis of commonly responding pathways revealed a conserved metabolic reprogramming pattern under high nitrogen supply. Commonly upregulated pathways were mainly enriched in Tryptophan metabolism and the Biosynthesis of tropane, piperidine, and isoquinoline alkaloids, indicating both nitrogen forms promote the synthesis of nitrogen-intensive defense compounds (**Figure 4A**). Concurrently, several lipid-related pathways (e.g., Fatty acid metabolism, Cutin, suberine and wax biosynthesis) were co-activated, suggesting enhanced cell membrane and barrier structures (**Figure 4A**). In contrast, the Flavonoid and isoflavonoid biosynthesis pathway was consistently and significantly suppressed, along with a weakening of some amino acid metabolism pathways (**Figure 4A**). This “promote defense, suppress symbiosis” pattern indicates that regardless of nitrogen form, high external nitrogen triggers a conserved resource trade-off strategy: metabolic resources are reallocated from the synthesis of symbiotic signaling molecules (flavonoids/isoflavonoids) to pathways related to direct chemical defense and structural integrity. This provides a metabolic network-level explanation for the conserved phenomenon of nitrogen induced inhibition of nodulation in soybean.

For DAMs specifically responding to ammonium, KEGG enrichment analysis showed that upregulated pathways were significantly enriched in multiple core metabolic processes, including nitrogen assimilation and amino acid metabolism (e.g., Nitrogen metabolism, Alanine, aspartate and glutamate metabolism, Arginine and proline metabolism), energy and nucleotide metabolism (e.g., Purine and pyrimidine metabolism), and secondary metabolite/cofactor biosynthesis (**Figure 4B**). In stark contrast, downregulated pathways were highly specifically enriched in the Flavonoid biosynthesis pathway (**Figure 4B**). These results indicate that ammonium treatment drives the activation of a metabolic network centered on efficient nitrogen assimilation and primary biosynthesis, while specifically inhibiting the flavonoid synthesis pathway closely associated with rhizobial symbiotic signaling. This explains, at the pathway level, why ammonium promotes plant growth while strongly inhibiting nodulation.

KEGG enrichment analysis of DAMs specifically responding to nitrate revealed an asymmetric metabolic reprogramming characterized by selective activation of defense-related secondary metabolic pathways and broad suppression of primary carbon and energy metabolism. Nitrate significantly upregulated pathways involved in defense and secondary metabolism, including Glucosinolate biosynthesis, alpha-Linolenic acid metabolism, 2-Oxocarboxylic acid metabolism, and Biosynthesis of secondary metabolites (**Figure 4C**). Glucosinolate biosynthesis is a core pathway for sulfur-containing defense compounds, while alpha-Linolenic acid metabolism is directly linked to jasmonic acid synthesis. In stark contrast, nitrate induced widespread downregulation of pathways central to carbohydrate metabolism, energy production, and primary biosynthesis, including sugar metabolism (e.g., Biosynthesis of nucleotide sugars, Starch and sucrose metabolism, Amino sugar metabolism), central carbon metabolism (e.g., Pentose phosphate pathway, Pyruvate metabolism), amino acid metabolism (e.g., Biosynthesis of amino acids, Alanine/aspartate/glutamate metabolism, Lysine biosynthesis), and other basal metabolic networks (e.g., Terpenoid backbone biosynthesis, Porphyrin metabolism, ABC transporters) (**Figure 4C**). These results indicate that nitrate drives a metabolic shift away from growth-oriented primary metabolism toward defense-related secondary metabolism, prioritizing chemical defense over biomass accumulation.

These pathway−level alterations elucidate the mechanism by which both nitrogen forms inhibit nodulation via suppression of symbiosis−related flavonoids, whereas nitrate exerts a stronger effect by concurrently activating defense pathways and suppressing primary metabolism, thereby creating a metabolic environment unfavorable for nodule formation yet supportive of root structural reinforcement.

### 3.5 Regulation of key metabolites and genes in the nitrogen metabolism pathway by different nitrogen sources

To elucidate the integrated regulatory mechanism linking the aforementioned metabolite accumulation and gene expression changes, and to clarify how different nitrogen sources systematically affect soybean nitrogen assimilation, we integrated metabolomic and transcriptomic data for a comprehensive analysis of the nitrogen metabolism pathway.

**Figure 5:**
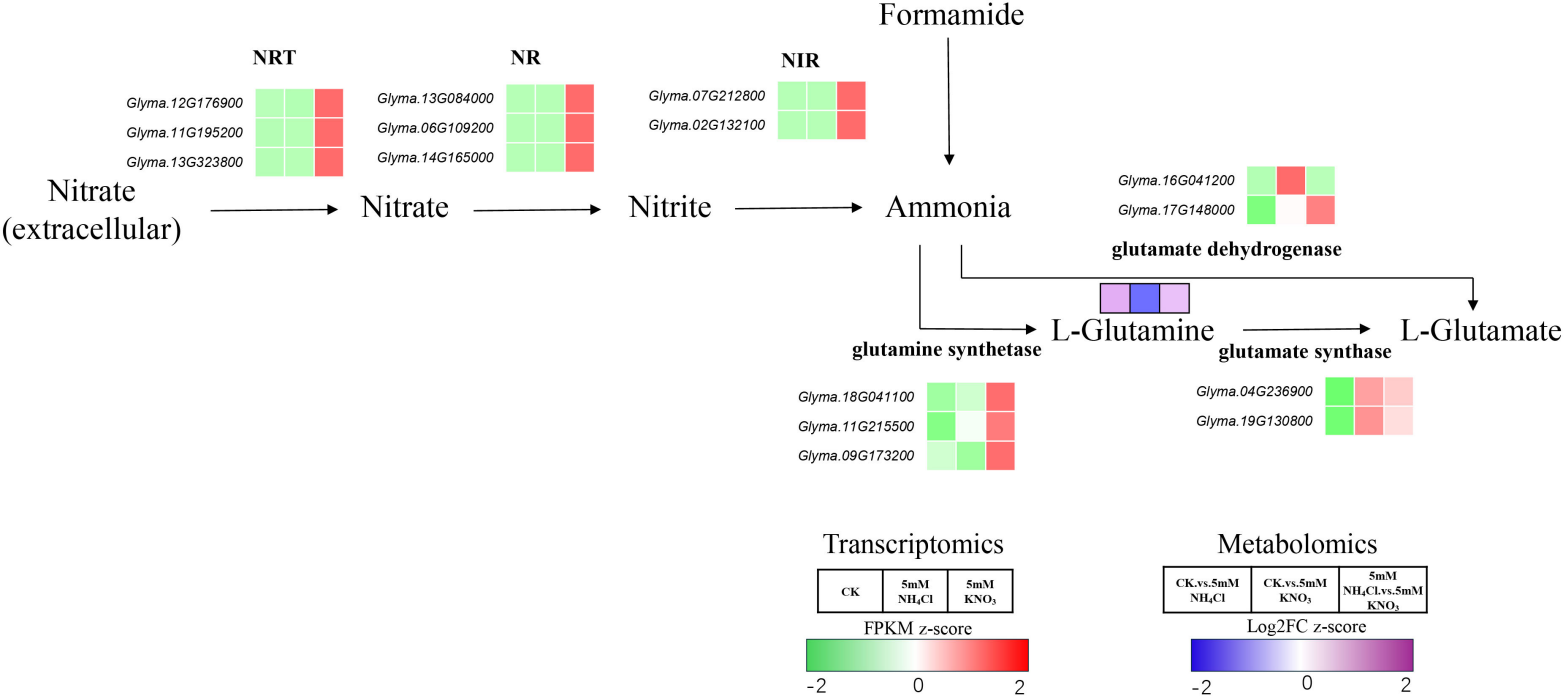
Schematic of key metabolite and gene expression changes in the soybean nitrogen metabolism pathway under different nitrogen sources. This schematic illustrates the transcriptomic (FPKM z-score heatmaps; color scale: −2 to 2, green–white–red) and metabolomic (Log_2_FC z-score heatmaps; color scale: −2 to 2, blue–white–purple) responses of core nitrogen metabolism components under three treatments: control (CK), 5 mM NH_4_Cl, and 5 mM KNO_3_. Key steps include: (1) nitrate uptake (NRT) and reduction (NR/NIR) to ammonia; (2) ammonia assimilation into L-glutamine/L-glutamate via GS/GOGAT or GDH; (3) ammonia generation from formamide. Red/purple indicates upregulated gene expression or increased metabolite accumulation, while green/blue represents downregulated expression or decreased accumulation.

Nitrogen assimilation begins with the transmembrane transport of extracellular nitrate, mediated by nitrate transporters (NRTs). Under nitrate treatment, the expression of several NRT genes (e.g., *Glyma.12G176900*, *Glyma.11G195200*, *Glyma.13G323800*) was specifically induced, with no significant changes under ammonium treatment (**Figure 5**). This differential expression pattern indicates that nitrate can enhance the efficiency of extracellular nitrate influx by activating NRT gene transcription. Intracellular nitrate is sequentially reduced to ammonia by nitrate reductase (NR) and nitrite reductase (NIR). The expression of several NR genes (e.g., *Glyma.13G084000*, *Glyma.06G109200*) and NIR genes (e.g., *Glyma.07G212800*, *Glyma.02G132100*) was significantly enhanced only under nitrate treatment, with no obvious change under ammonium treatment (**Figure 5**). These results demonstrate that the NR- and NIR-mediated nitrate reduction process features nitrate-dependent activation.

Ammonia assimilation is the core node of nitrogen metabolism, with its product L-glutamine being a key molecule for nitrogen transport and redistribution in plants. Metabolomic data showed a significant increase in L-glutamine under ammonium treatment, suggesting ammonium-preferential accumulation. Among the genes encoding corresponding assimilation enzymes, glutamine synthase (GS, e.g., *Glyma.18G041100, Glyma.11G215500*) was significantly upregulated under nitrate treatment compared with both CK and ammonium (NH_4_Cl) treatment, and its expression level under nitrate was much higher than that under ammonium. Glutamate synthase (GOGAT, e.g., *Glyma.04G236900, Glyma.19G130800*) genes were upregulated under both ammonium and nitrate treatments relative to CK, but their expression levels were slightly higher under ammonium than under nitrate, indicating that GOGAT-mediated ammonia assimilation is activated by both nitrogen sources but exhibits differential expression intensity. In contrast, glutamate dehydrogenase (GDH, e.g., *Glyma.16G041200*, *Glyma.17G148000*) exhibited isoform-specific expression patterns: *Glyma.16G041200* was specifically upregulated under ammonium treatment but downregulated under nitrate treatment, while *Glyma.17G148000* showed significant upregulation under nitrate treatment. This indicates that GDH-mediated ammonia assimilation is regulated in an isoform-specific manner rather than a uniform ammonium-specific response (**Figure 5**).

In summary, different nitrogen sources systematically regulate soybean nitrogen assimilation through distinct regulatory strategies targeting key steps of the nitrogen metabolism pathway. Nitrate specifically activates the entire nitrate-dependent assimilation process, including the transcription of NRT, NR, and NIR genes, to promote nitrate uptake and reduction to ammonia, while also inducing high-level expression of GS genes. Ammonium, by contrast, does not affect nitrate transport and reduction, but promotes L-glutamine accumulation and modulates ammonia assimilation through differential regulation of GOGAT and GDH isoforms. Collectively, these findings clarify the nitrogen source-specific regulatory networks governing soybean nitrogen assimilation, highlighting the divergent molecular mechanisms by which ammonium and nitrate orchestrate nitrogen metabolism. The contrasting regulatory strategies of nitrate and ammonium in nitrogen assimilation help explain their phenotypic outcomes: ammonium−driven glutamine accumulation supports root biomass production, whereas nitrate−induced NR/NIR activation increases metabolic cost and oxidative pressure, conditions known to suppress nodulation.

### 3.6 Response of flavonoid and isoflavonoid metabolic pathways to different nitrogen sources

Flavonoids and isoflavonoids play crucial roles in plant-rhizobia symbiotic nitrogen fixation. As important plant secondary metabolites, flavonoids act as signal molecules promoting rhizobial infection and nodule development, scavenge reactive oxygen species to protect both plant and rhizobia from oxidative damage, thereby maintaining symbiotic stability. Isoflavonoids, a subclass of flavonoids, can activate nodulation genes and regulate symbiosis specificity (Dakora and Phillips, 1996; Winkel-Shirley, 2001; Hassan and Mathesius, 2012; García-Calderón et al., 2020).

**Figure 6:**
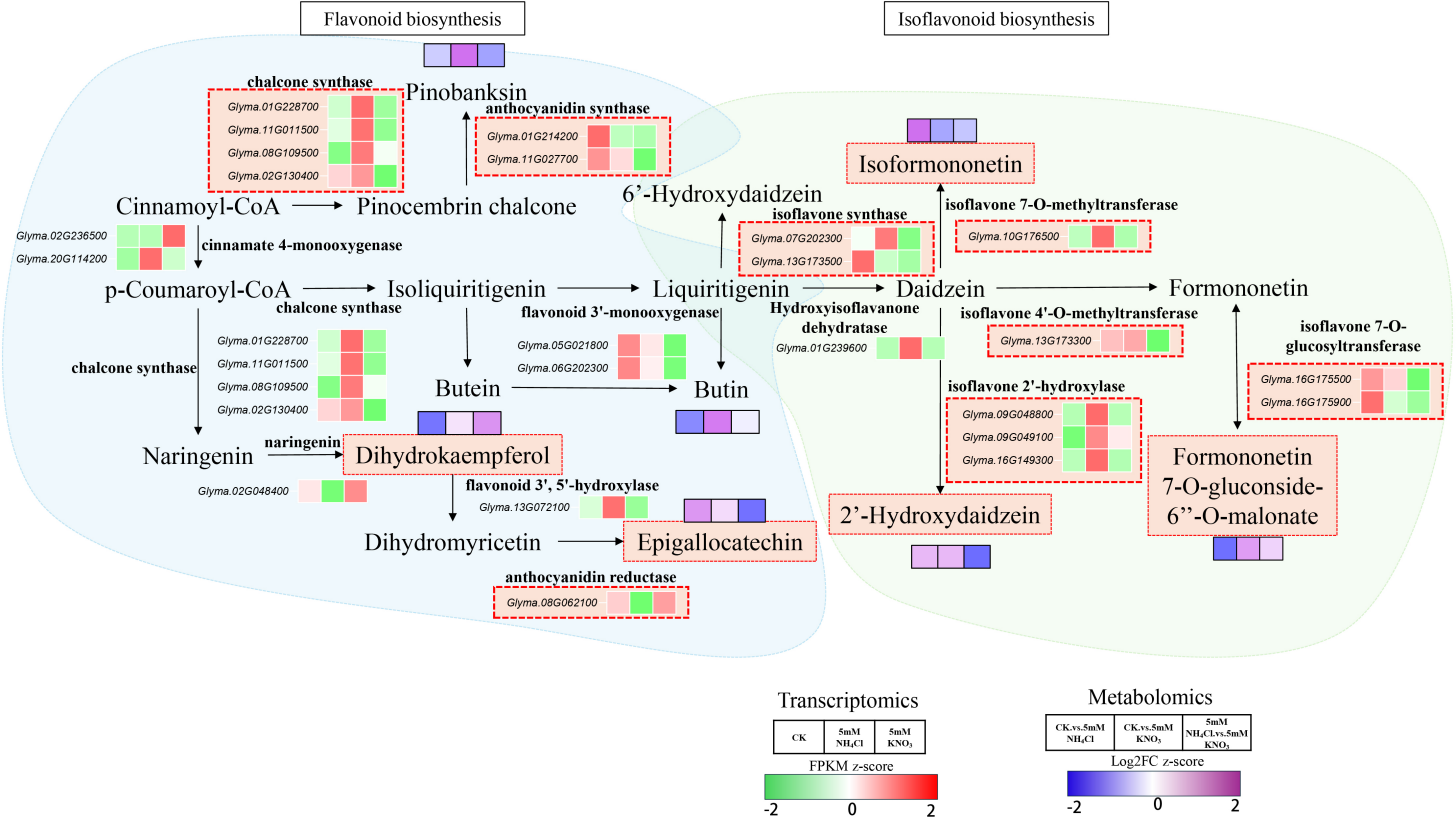
Differential responses of the flavonoid and isoflavonoid biosynthetic pathways to nitrate and ammonium in soybean. This schematic shows the expression of key biosynthetic genes (transcriptomics, FPKM z-score; color scale: −2 to 2, green–white–red) and accumulation of corresponding metabolites (metabolomics, Log_2_FC z-score; color scale: −2 to 2, blue–white–purple) in soybean under three treatments: control (CK), 5 mM NH_4_Cl, and 5 mM KNO_3_. The flavonoid pathway (left) is marked with a light blue background, and the isoflavonoid pathway (right) with a light green background. Red/purple indicates upregulation/increased accumulation, while green/blue indicates downregulation/decreased accumulation. Core genes and metabolites enclosed in red dotted boxes show nitrogen-form-specific responses, including opposite trends between ammonium and nitrate or decoupling between transcript and metabolite levels.

Metabolomic analysis revealed nitrogen−form−specific accumulation patterns of flavonoid and isoflavonoid metabolites. Dihydrokaempferol was downregulated under ammonium (NH_4_Cl) but upregulated under nitrate (KNO_3_), resulting in higher levels under nitrate. In contrast, Epigallocatechin and 2’−Hydroxydaidzein were induced by both nitrogen sources but accumulated to higher levels under ammonium. Isoformononetin was enhanced by ammonium yet suppressed by nitrate, whereas Formononetin 7−O−glucoside−6’’−O−malonate showed the opposite trend, being downregulated by ammonium and upregulated by nitrate. These patterns indicate divergent pathway regulation: ammonium preferentially promotes Epigallocatechin, 2′−Hydroxydaidzein, and Isoformononetin accumulation, whereas nitrate drives Dihydrokaempferol and Formononetin 7−O−glucoside−6’’−O−malonate production (**Figure 6**).

Integrating these metabolite profiles with transcriptomic data reveals a clear divergence between flavonoid and isoflavonoid pathway regulation. In the flavonoid pathway, ammonium treatment induced higher expression of upstream chalcone synthase genes (CHS, e.g., *Glyma.01G228700*, *Glyma.11G011500*), consistent with the enhanced accumulation of Epigallocatechin and 2’−Hydroxydaidzein. In contrast, nitrate treatment upregulated downstream branch genes such as anthocyanidin synthase (ANS, e.g., *Glyma.01G214200*, *Glyma.11G027700*) and anthocyanidin reductase (ANR, e.g., *Glyma.08G062100*), aligning with the nitrate−preferential accumulation of Dihydrokaempferol.

By comparison, the isoflavonoid pathway exhibited a markedly different transcriptional pattern. All major biosynthetic genes—including isoflavone synthase (IFS, e.g., *Glyma.07G202300*, *Glyma.13G173500*), isoflavone 7−O−methyltransferase (e.g., *Glyma.10G176500*), isoflavone 4’−O−methyltransferase (e.g., *Glyma.13G173300*), isoflavone 7−O−glucosyltransferase (e.g., *Glyma.16G175500*, *Glyma.16G175900*), and isoflavone 2’−hydroxylase (e.g., *Glyma.09G048800*, *Glyma.09G049100*, *Glyma.16G149300*) were significantly downregulated under nitrate relative to ammonium. This transcriptional suppression corresponds with the reduced accumulation of Isoformononetin under nitrate. However, the nitrate−enhanced accumulation of Formononetin 7−O−glucoside−6’’−O−malonate occurred despite the downregulation of its upstream biosynthetic genes, indicating a decoupling between transcript abundance and metabolite output.

Together, these results demonstrate that ammonium and nitrate impose distinct regulatory architectures on flavonoid and isoflavonoid biosynthesis. Ammonium preferentially enhances the production of Epigallocatechin, 2′−Hydroxydaidzein, and Isoformononetin through higher expression of upstream flavonoid and isoflavonoid genes, whereas nitrate promotes Dihydrokaempferol and Formononetin 7−O−glucoside−6″−O−malonate accumulation despite transcriptional repression of most isoflavonoid pathway genes, suggesting additional layers of metabolic control. Because flavonoids and isoflavonoids are essential for rhizobial recognition and infection thread formation, their nitrogen−form−specific suppression provides a direct mechanistic explanation for the reduced nodulation observed under both treatments, particularly under nitrate.

### 3.7 Different nitrogen sources influence arginine biosynthesis

Arginine is an important amino acid that, besides participating in protein synthesis and the urea cycle, serves as a precursor for signaling molecules like nitric oxide and polyamines. These can promote adventitious and lateral root formation by regulating auxin pathways, thereby enhancing root expansion and the absorption of water and nutrients (Winter et al., 2015; Cruz et al., 2006; Chen et al., 2022).

**Figure 7:**
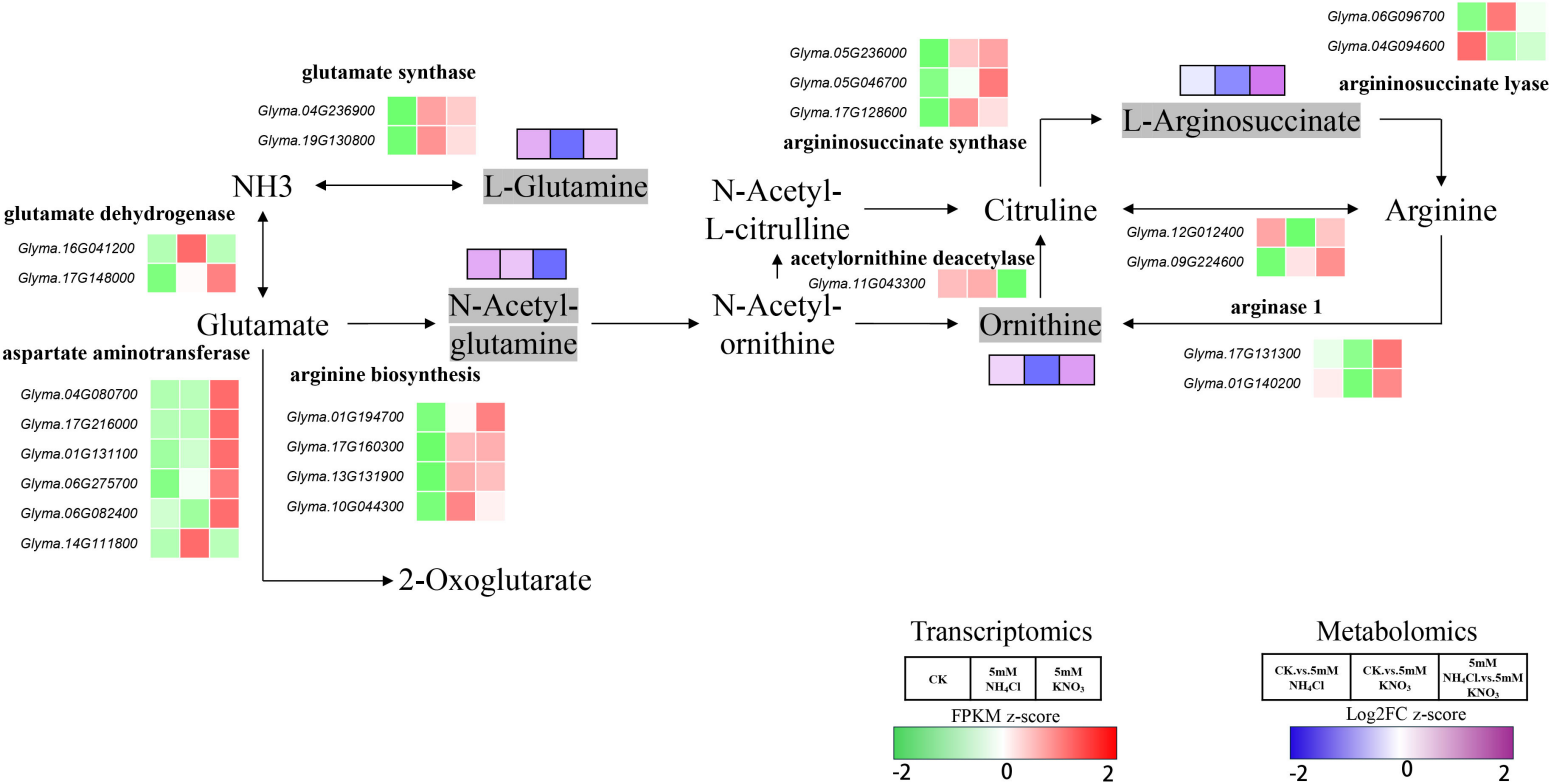
Integrative analysis of the arginine biosynthesis pathway in soybean roots under different nitrogen sources. This schematic shows the combined transcriptomic (FPKM z-score; color scale: −2 to 2, green–white–red) and metabolomic (Log_2_FC z-score; color scale: −2 to 2, blue–white–purple) responses of the arginine biosynthesis pathway and related ammonia assimilation in soybean roots under three treatments: CK, 5 mM NH_4_Cl, and 5 mM KNO_3_. Key steps: (1) Ammonia assimilation into L-glutamate/L-glutamine via GDH and GS-GOGAT cycle; (2) L-glutamate sequential conversion to arginine; (3) Arginine catabolism to ornithine via arginase 1. Red/purple indicates upregulation/increased accumulation, while green/blue indicates downregulation/decreased accumulation.

Under ammonium treatment, glutamate dehydrogenase (GDH, e.g., *Glyma.16G041200*, *Glyma.17G148000*) and glutamate synthase (GOGAT, *Glyma.04G236900*, *Glyma.19G130800*) were significantly upregulated, activating the glutamate synthesis pathway. Meanwhile, most aspartate aminotransferase (AAT, e.g., *Glyma.04G080700*, *Glyma.17G216000*, *Glyma.01G131100*, *Glyma.06G275700*, *Glyma.06G082400*) genes were downregulated, with only *Glyma.14G111800* being induced. Metabolomic results showed that L-Glutamine, N-Acetyl-L-glutamic acid, and L-Ornithine were all significantly accumulated, while Argininosuccinic acid only slightly decreased, indicating that nitrogen flux was preferentially directed to glutamate and the acetylation branch, promoting ornithine synthesis. In the acetylation module, genes involved in arginine biosynthesis (e.g., *Glyma.01G194700*, *Glyma.17G160300*, *Glyma.13G131900*, *Glyma.10G044300*) and acetylornithine deacetylase (ARGD, e.g., *Glyma.11G043300*) were significantly upregulated, further facilitating ornithine production. Although the expression of arginase 1 (ARG1, e.g., *Glyma.17G131300*, *Glyma.01G140200*) was inhibited, the overall activation of the acetylation branch still led to an increase in ornithine content (**Figure 7**).

In contrast, under nitrate treatment, most AAT genes (e.g., *Glyma.04G080700*, *Glyma.17G216000*, *Glyma.01G131100*, *Glyma.06G275700*, *Glyma.06G082400*) were significantly upregulated, the glutamate cycle mediated by transamination was significantly enhanced, and only *Glyma.14G111800* was downregulated. In the arginine synthesis branch, argininosuccinate synthase (ASS, e.g., *Glyma.05G236000*, *Glyma.05G046700*, *Glyma.17G128600*) was generally upregulated, while argininosuccinate lyase (ASL, e.g., *Glyma.06G096700*, *Glyma.04G094600*) was downregulated. Metabolomic analysis showed that Argininosuccinic acid was significantly reduced, suggesting that the transcriptional upregulation of ASS did not translate into metabolite accumulation, with regulatory limitations at the post-transcriptional or enzymatic activity level. At the same time, both isoforms of ARG1 (e.g., *Glyma.17G131300*, *Glyma.01G140200*) were strongly induced, accelerating arginine degradation. However, acetylornithine deacetylase (*Glyma.11G043300*), a key enzyme in the acetylation branch, was significantly downregulated, directly limiting ornithine synthesis. Metabolomic results confirmed that both L-Ornithine and L-Glutamine were significantly decreased, indicating that the weakened synthesis capacity of the acetylation branch dominated the net reduction of ornithine (**Figure 7**).

Collectively, ammonium promoted the accumulation of L-Glutamine, N-Acetyl-L-glutamic acid, and ornithine by activating the GDH/GOGAT pathway and the acetylation branch. In contrast, nitrate caused a decrease in Argininosuccinic acid, ornithine, and L-Glutamine contents through isoform-specific gene regulation, forming distinct arginine metabolic patterns. These nitrogen−dependent shifts in arginine metabolism influence the production of polyamines and nitric−oxide−related intermediates, which are key regulators of root branching and nodule organogenesis, thereby linking metabolic changes to the contrasting effects of ammonium and nitrate on root development and nodulation.

## 4 Discussion

Nitrogen form is a decisive environmental cue that shapes the balance between symbiosis and root development in legumes. By integrating phenotypic, metabolomic, and transcriptomic datasets, this study demonstrates that ammonium and nitrate impose distinct yet partially overlapping regulatory programs on soybean roots. Although both nitrogen forms strongly inhibited nodulation, nitrate exerted a more pronounced effect while driving disproportionate root system expansion. These findings align with classical observations that high nitrogen suppresses nodulation and extend them by showing that nitrogen form—not merely nitrogen level—is the dominant determinant of the symbiosis–growth trade−off.

### 4.1 Nitrogen form drives a strategic shift between symbiosis and autonomous growth

Both ammonium and nitrate strongly inhibited nodulation, consistent with long−standing observations that high nitrogen suppresses symbiotic nitrogen fixation (Streeter, 1986; Hardarson et al., 1989; Sulieman et al., 2014). However, the magnitude and metabolic context of inhibition differed sharply between nitrogen forms. Nitrate imposed a far stronger suppression of nodulation while simultaneously promoting extensive root proliferation, whereas ammonium induced a more moderate inhibition accompanied by enhanced primary metabolism. The contrasting responses support the view that nitrogen form determines whether the plant allocates resources toward symbiotic investment or autonomous growth. Ammonium appears to signal a nutrient−rich and microbially active environment that favors rapid growth, whereas nitrate conveys an oxidized and disturbance−prone environment in which defense and resource storage become advantageous. This ecological interpretation provides a conceptual framework for understanding why nitrogen form exerts such strong and divergent effects on legume development. Together, these nitrogen−dependent shifts clarify the phenotypic outcomes observed in this study: nodulation is reduced because investment in symbiotic signaling and infection processes declines, while root system expansion is promoted through the redirection of metabolic resources toward independent growth and structural development.

### 4.2 Metabolic network reprogramming reveals nitrogen−form−specific regulatory logic

The identification of six metabolic modules highlights the modularity and plasticity of the soybean metabolic network. Modules shared between ammonium and nitrate reflect a conserved high−nitrogen response characterized by suppression of flavonoid/isoflavonoid biosynthesis and activation of defense−related pathways. This conserved shift away from symbiotic signaling toward chemical defense provides a metabolic explanation for nitrogen−induced nodulation inhibition (Dakora and Phillips, 1996; Hassan and Mathesius, 2012).

In contrast, nitrogen−form−specific modules reveal distinct regulatory logics: ammonium enhanced nitrogen assimilation and primary metabolic flux, whereas nitrate activated diverse secondary metabolites, lipid remodeling, and redox−associated pathways. These findings support a metabolic−context hypothesis, in which nitrogen form determines the metabolic “background state” that shapes downstream physiological outcomes. The nitrogen−form−specific modules therefore provide a mechanistic bridge between metabolic state and developmental outcomes: modules enriched for flavonoid suppression correspond to impaired rhizobial recognition and nodule initiation, while modules enriched for nitrogen assimilation or lipid remodeling align with the enhanced root biomass and architectural changes observed under ammonium and nitrate, respectively.

Future studies should dissect the transcriptional regulators and signaling molecules that define these modules, particularly nitrogen−form−specific transcription factors and redox−responsive regulators.

### 4.3 Nitrogen assimilation is shaped by carbon economy rather than transcription alone

Although nitrate strongly induced the NRT–NR–NIR cascade and GS expression, glutamine levels declined under nitrate treatment. This decoupling suggests that nitrogen assimilation is constrained by carbon availability and energy cost, not transcriptional activation alone. Nitrate reduction requires substantial reductant and carbon skeletons (Masclaux−Daubresse et al., 2010; Xu et al., 2012), which may limit amino acid synthesis and redirect carbon toward defense metabolism.

We propose a carbon−cost hypothesis, in which nitrate imposes a metabolic burden that forces the plant to prioritize defense and structural reinforcement over symbiotic investment. This carbon−cost constraint provides a direct explanation for the phenotypic divergence: nitrate−driven metabolic burden limits the carbon available for nodule organogenesis and infection thread formation, while simultaneously promoting root thickening and elongation as compensatory growth. In contrast, ammonium’s lower assimilation cost supports both nitrogen incorporation and moderate root expansion without imposing strong inhibitory pressure on nodulation.

### 4.4 Flavonoid and isoflavonoid pathways act as a central metabolic hub for nitrogen-form perception

Flavonoids and isoflavonoids are essential for rhizobial recognition, oxidative stress mitigation, and nitrogen metabolism (Dixon & Pasinetti, 2010; García-Calderón et al., 2020). Our data reveal that ammonium and nitrate suppress these pathways through distinct mechanisms: ammonium primarily affects upstream CHS-mediated flux, whereas nitrate suppresses downstream isoflavonoid branches while activating ANS/ANR.

These findings support a hub−and−switch model, in which the flavonoid/isoflavonoid network serves as a central metabolic hub integrating nitrogen signals, while ammonium and nitrate act as distinct regulatory switches that modulate different nodes of the pathway. Because flavonoids and isoflavonoids are indispensable for rhizobial attraction, Nod−factor signaling, and early nodule organogenesis, their nitrogen−form−specific suppression provides a direct mechanistic basis for the reduced nodulation observed in our phenotypic data. The differential targeting of upstream versus downstream branches also explains why ammonium and nitrate produce distinct metabolite signatures yet converge on nodulation inhibition.

Future work should identify the transcription factors and signaling molecules that mediate nitrogen−form−specific regulation of this hub.

### 4.5 Arginine metabolism integrates nitrogen form with root developmental outcomes

Arginine metabolism emerged as a nitrogen−form−sensitive signaling axis. Ammonium enhanced ornithine turnover and likely promoted polyamine biosynthesis, which is known to stimulate root proliferation (Cruz et al., 2006). Nitrate increased arginine accumulation and ARG1 expression, potentially enhancing nitric oxide (NO) production—a key regulator of root meristem activity and lateral root patterning (Correa-Aragunde et al., 2004).

We propose an arginine−signal partitioning hypothesis, in which nitrogen form determines whether arginine is directed toward polyamine−mediated growth signals or NO−mediated developmental remodeling. These nitrogen−dependent shifts in arginine partitioning therefore offer a mechanistic explanation for the contrasting root architectures observed: ammonium promotes polyamine−mediated root proliferation and lateral root formation, whereas nitrate enhances NO−mediated developmental remodeling, contributing to thicker, more elongated root systems. Testing this model will require genetic manipulation of ASS, ARGD, and ARG1, as well as pharmacological modulation of NO and polyamine pathways.

### 4.6 A unified conceptual model for nitrogen−form−dependent developmental decisions

Integrating these findings, we propose that nitrogen form functions as a developmental decision signal that coordinates carbon–nitrogen allocation, metabolic network topology, and the balance between symbiosis and autonomous growth. Ammonium drives a growth−oriented program characterized by efficient nitrogen assimilation and polyamine−linked root expansion, whereas nitrate induces a defense−adaptation program characterized by redox−associated metabolism, NO signaling, and strong nodulation inhibition.

This unified model integrates metabolic, transcriptional, and phenotypic evidence to explain how nitrogen form dictates the balance between nodulation and root development. By determining whether resources flow toward symbiotic organogenesis or autonomous root expansion, nitrogen form acts as a developmental switch that shapes the overall architecture and nitrogen−use strategy of soybean. Future genetic studies targeting CHS, IFS, ASS, ARG1, and key nitrogen−responsive transcription factors will be essential to validate and refine this framework.

## 5 Conclusion

This study demonstrates that nitrogen form serves as a key developmental signal that reshapes the balance between nodulation and root growth in soybean. Although both ammonium and nitrate suppress nodule formation, the two nitrogen sources trigger distinct metabolic states that lead to divergent developmental outcomes: ammonium supports a growth−oriented state characterized by efficient ammonia assimilation and polyamine−linked root expansion, whereas nitrate induces a defense− and remodeling−oriented state that strongly inhibits early symbiotic signaling and promotes extensive root architectural changes. Multi−omics integration revealed that nitrogen form shapes the metabolic landscape underlying developmental decisions by differentially regulating nitrogen assimilation pathways, flavonoid/isoflavonoid biosynthesis, and arginine partitioning, and such coordinated metabolic alterations elucidate how plants prioritize carbon and nitrogen allocation among symbiosis, growth, and defense under distinct nitrogen regimes. Together, our findings provide a concise transcriptomic and metabolomic mechanistic framework for understanding nitrogen−form−dependent developmental decisions in legumes and offer a conceptual basis for optimizing nitrogen management and symbiotic efficiency in soybean cultivation.

## Data Availability

The original contributions presented in the study are included in the article/**Supplementary Material**. Further inquiries can be directed to the corresponding authors.

## Supplementary material

Supplementary Figure 1 Volcano plot analysis of metabolites in soybean roots under different nitrogen treatments. The distribution of differentially accumulated metabolites across three pairwise comparisons: CK vs. 5 mM NH_4_Cl, CK vs. 5 mM KNO_3_, and 5 mM NH_4_Cl vs. 5 mM KNO_3_. The x-axis represents log_2_(Fold Change), and the y-axis represents −log_10_(P-value). Red dots indicate significantly upregulated metabolites, green dots indicate significantly downregulated metabolites, and gray dots represent metabolites with no significant differences.

Supplementary Figure 2 Venn diagram of differentially accumulated metabolites in soybean roots under different nitrogen treatments. Venn diagram illustrating the overlap of DAMs across three comparisons: CK vs. 5 mM NH_4_Cl, CK vs. 5 mM KNO_3_, and 5 mM NH_4_Cl vs. 5 mM KNO_3_.

Supplementary Figure 3 KEGG pathway enrichment analysis of all differentially accumulated metabolites in soybean roots under different nitrogen forms. Pathway enrichment of all DAMs across three comparisons: 5 mM NH_4_Cl.vs. 5 mM KNO_3_, 5 mM NH_4_Cl.vs. CK, and 5 mM KNO_3_.vs. CK. X-axis: Differential Abundance (DA) Score (positive values indicate upregulation, negative values indicate downregulation). The Y−axis represents pathway names. Dot color indicates the P−value, with redder colors representing higher significance. Dot size represents the number of DAMs in each pathway.
