# Supplementary figures and images for: Multi-omics reveals metabolic reprogramming underlying differential modulation of nodulation and root development by nitrate and ammonium in soybean
Source: Front Plant Sci. 2026 Apr 22;17:1813591. doi: 10.3389/fpls.2026.1813591 (PMC13144147; doi:10.3389/fpls.2026.1813591)

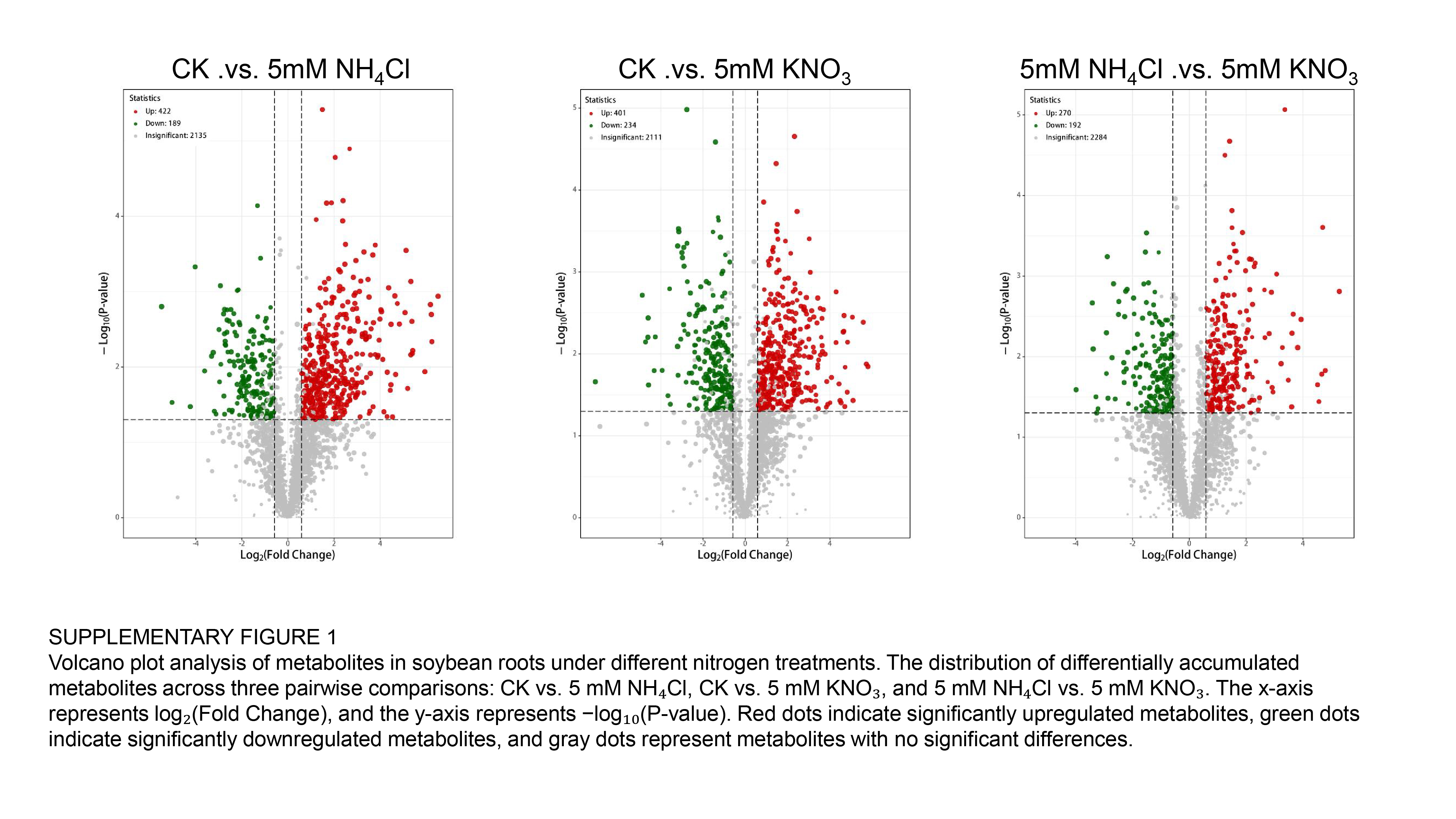

Supplement: Supplementary Figure 1 — Volcano plot analysis of metabolites in soybean roots under different nitrogen treatments. The distribution of differentially accumulated metabolites across three pairwise comparisons: CK vs. 5 mM NH4Cl, CK vs. 5 mM KNO3, and 5 mM NH4Cl vs. 5 mM KNO3. The x-axis represents log2(Fold Change), and the y-axis represents −log10(P-value). Red dots indicate significantly upregulated metabolites, green dots indicate significantly downregulated metabolites, and gray dots represent metabolites with no significant differences. [file Image1.jpeg]

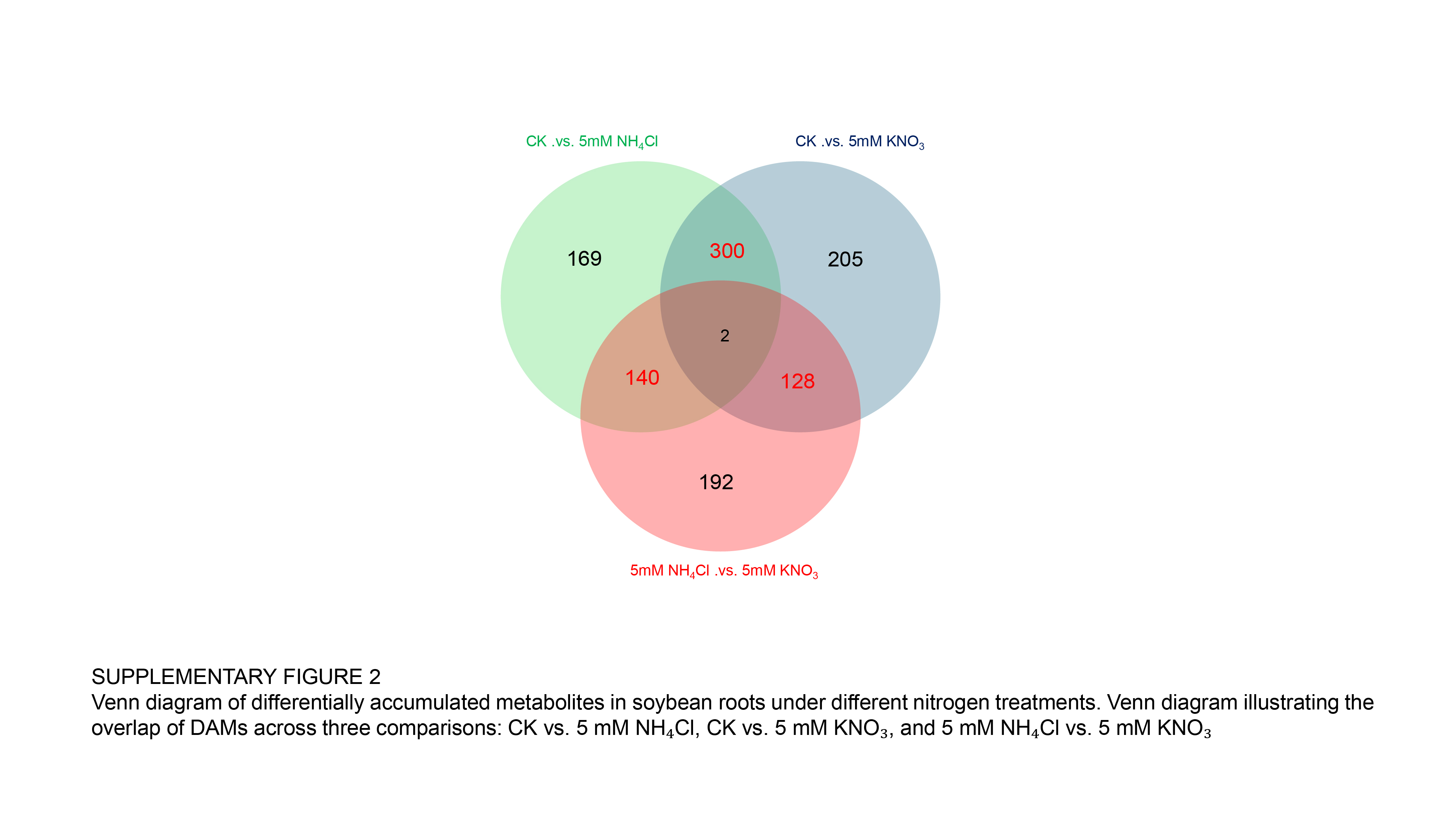

Supplement: Supplementary Figure 2 — Venn diagram of differentially accumulated metabolites in soybean roots under different nitrogen treatments. Venn diagram illustrating the overlap of DAMs across three comparisons: CK vs. 5 mM NH4Cl, CK vs. 5 mM KNO3, and 5 mM NH4Cl vs. 5 mM KNO3. [file Image2.jpeg]

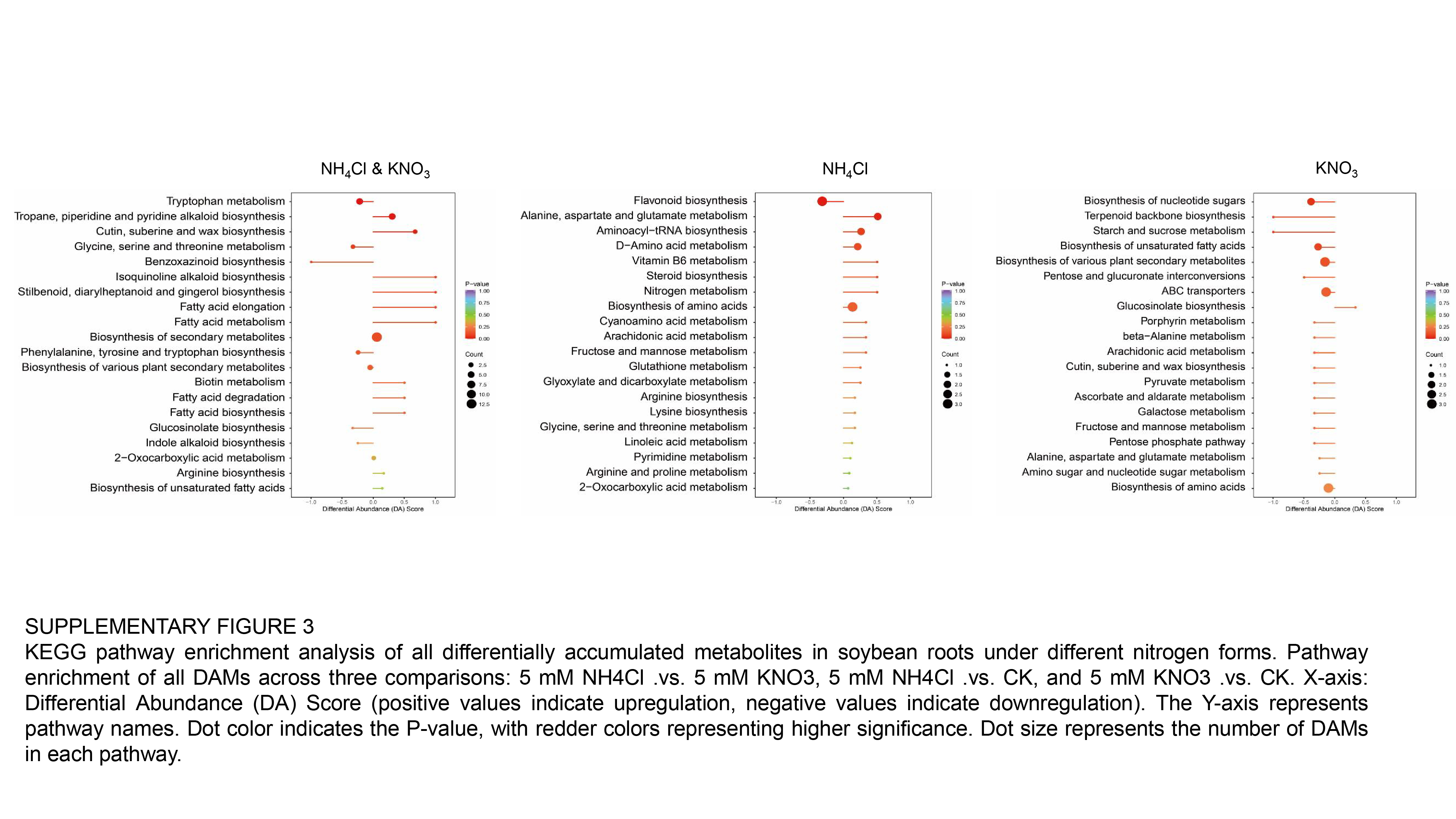

Supplement: Supplementary Figure 3 — KEGG pathway enrichment analysis of all differentially accumulated metabolites in soybean roots under different nitrogen forms. Pathway enrichment of all DAMs across three comparisons: 5 mM NH4Cl.vs. 5 mM KNO3, 5 mM NH4Cl.vs. CK, and 5 mM KNO3.vs. CK. X-axis: Differential Abundance (DA) Score (positive values indicate upregulation, negative values indicate downregulation). The Y−axis represents pathway names. Dot color indicates the P−value, with redder colors representing higher significance. Dot size represents the number of DAMs in each pathway. [file Image3.jpeg]
